# Effectiveness of Community Pharmacy Diabetes and Hypertension Care Program: An Unexplored Opportunity for Community Pharmacists in Pakistan

**DOI:** 10.3389/fphar.2022.710617

**Published:** 2022-05-17

**Authors:** Madeeha Malik, Azhar Hussain, Usman Aslam, Ayisha Hashmi, Mojtaba Vaismoradi, Khezar Hayat, Shazia Jamshed

**Affiliations:** ^1^ Department of Pharmacy Practice, Hamdard Institute of Pharmaceutical Sciences, Hamdard University Islamabad, Islamabad, Pakistan; ^2^ Faculty of Nursing and Health Sciences, Nord University, Bodø, Norway; ^3^ Institute of Pharmaceutical Sciences, University of Veterinary and Animal Sciences, Lahore, Pakistan; ^4^ Department of Clinical Pharmacy and Practice, Faculty of Pharmacy, Universiti Sultan Zainal Abidin, Terengganu, Malaysia; ^5^ Qualitative Research-Methodological Application in Health Sciences Research Group, Kulliyyah of Pharmacy, International Islamic University Malaysia, Kuantan, Malaysia

**Keywords:** diabetes, hypertension, blood pressure, community pharmacist, Pakistan

## Abstract

**Background:** The effective management of patients diagnosed with both Diabetes as well as Hypertension is linked with administration of efficacious pharmacological therapy as well as improvement in adherence through counseling and other strategies. Being a part of primary healthcare team, community pharmacists can effectively provide patient care for chronic disease management. The objective of the study was to evaluate the impact of pharmacist counseling on blood pressure and blood glucose control among patients having both hypertension and diabetes attending community pharmacies in Pakistan.

**Method:** A randomized, controlled, single-blind, pre-post-intervention study design was used. The respondents included patients diagnosed with diabetes mellitus (Type I or II) and hypertension visiting community pharmacies to purchase their regular medicine. A simple random sampling technique using the lottery method was used to select community pharmacies in groups A (intervention, *n* = 4) and group B (control, *n* = 4). The total number of patients was 40 in each group, while estimating a dropout rate of 25%. The patients in the intervention group received special counseling. Blood pressure and blood glucose were checked after every 15 days for 6 months. Prevalidated tools such as the hypertension knowledge level scale, the diabetes knowledge questionnaire 24, and a brief medication questionnaire was used. Data were coded and analyzed using SPSS 21. Wilcoxon test (*p* < 0.05) was used to compare pre-post intervention knowledge regarding the disease, while the Mann-Whitney test (*p* < 0.05) was used to find differences in medication adherence among control and intervention groups.

**Results:** A significant improvement in mean knowledge scores of patients with diabetes (16.02 ±2.93 vs. 19.97 ±2.66) and hypertension (15.60 ±3.33 vs. 18.35 ±2.31) in the intervention group receiving counseling for 6 months than control group (*p* < 0.05) was noted. Furthermore, the fasting blood glucose levels (8.25 ±1.45) and systolic BP (130.10 ±6.89) were significantly controlled after 6 months in the intervention group.

**Conclusion:** The current study results concluded that community pharmacists’ counselling has a positive impact on blood glucose and blood pressure management among patients suffering with both diabetes and hypertension.

## Introduction

Throughout the world, the burden of disease due to chronic metabolic disorders is rising at an alarming rate (Schultz et al., 2021). Diabetes mellitus and hypertension are the most common chronic and noncommunicable diseases that affect the majority of the population residing in developed and developing countries (Smith et al., 2021). The prolonged hyperglycemia as well as increased blood pressure is related with long-term impairment, dysfunction, and failure of different organs, including eyes, kidneys, nerves, heart, and blood vessels (Kwakye et al., 2021). Pakistan has been ranked third in the burden of diabetes with a prevalence of 33 million adults in 2021 (IDF, 2021). Furthermore, according to an estimate, every one in four adults is diagnosed with hypertension in Pakistan (Benedict et al., 2018). Various factors contribute to the ineffective management of diabetes and hypertension, including low health literacy, inadequate knowledge, and poor self-care behaviors. Poor blood glucose and blood pressure control have been reported in individuals with low health literacy and poor numeracy skills (Withidpanyawong et al., 2019). The chances of non-compliance to prescriber recommendations are increased due to dissatisfaction, poor relationship with patient, long waiting times, and high cost of follow-up visits. The use of multiple medications and increased frequency of drugs also leads to non-compliance in management of these chronic diseases. Furthermore, the absence of a cost-effective and friendly healthcare system is one of the main contributors to patient nonadherence to medication (Prudencio et al., 2018). Limited time and resources, inappropriate prescribing, improper assessment of patient needs, no proper goal setting and reduced motivation for patients are just a few of the barriers faced by physicians (Stanton-Robinson et al., 2018). Ineffective communication skills of health care professionals and lack of appropriate counseling and shared decision-making skills also contribute to failure of effective management of these chronic disorders (Milosavljevic et al., 2018).

The increasing incidence of diabetes mellitus and hypertension has emphasized the development and implementation of effective management programs for both diseases at the primary health care level. One such intervention suggests the participation of community pharmacists in the management of chronic diseases which has resulted in positive results in various healthcare settings (Godman et al., 2020). Community pharmacists can play an important role in the treatment of diabetes and hypertension by helping patients achieve their therapeutic and lifestyle goals (Machen et al., 2019). A study conducted in Japan reported that interventions focused on lifestyle modifications by community pharmacists improved glycemic control in patients with Type II diabetes mellitus. After 6 months of intervention, the patients had improved HbA1c levels, as well as the number of drugs used to control blood glucose was also reduced (Okada et al., 2016). As experts in drug therapy, drug selection, and patient education, community pharmacists can be excellent additions to the multidisciplinary primary health care team, contributing to better patient care. A study conducted in Canada showed that the addition of a pharmacist to the primary care team for the treatment of hypertension and type II diabetics can lead to improved blood pressure and glucose control through medication assessments, history and physical examinations and patient counseling services (Simpson et al., 2015). Despite new medications and specific care for patients with diabetes mellitus and hypertension, control of glycemic parameters, blood pressure, and lipid profile remains largely inadequate in Pakistan. Factors that include the absence of pharmacists at community pharmacies and the lack of appropriate counseling led to the irrational use of drugs (Hayat et al., 2019; Hayat et al., 2020). Therefore, the present study was designed to evaluate the impact of patient counseling by community pharmacists on blood pressure and glucose control among patients with diabetes and hypertension in twin cities of Pakistan.

## Method

### Study Design

A randomized, controlled, single-blind, pre-post-intervention study design was used to evaluate the impact of community pharmacist counseling on blood pressure and glucose control of patients attending community pharmacies in Pakistan who suffer from both hypertension and diabetes. Community pharmacies were randomly selected, which reduced the likelihood of selection and confounding bias in determining outcomes. Participants and community pharmacists were kept blinded on the allocation of participants to the control and intervention groups to reduce information bias. Study approval (ERC/HU 029) was obtained from the Ethics Committee of Hamdard University. Informed written consent was taken by all the community pharmacists from the patients willing to participate in the study.

### Study Site and Respondents

The study sites were community pharmacies located in twin cities (Islamabad and Rawalpindi) of Pakistan. The respondents included patients diagnosed with both diabetes mellitus (Type I or II) and hypertension visiting community pharmacies for purchasing their regular medicine.

### Inclusion Criteria and Exclusion Criteria

Patients diagnosed with diabetes mellitus (Type I or II) and hypertension with an HbA1c value ≥ 7% and BP greater than 140/90 mmHg at the time of diagnosis were included in the study. Patients diagnosed with any concurrent endocrine disorder (such as thyroid disorders, obesity, and gestational diabetes), cardiac heart failure, end-stage renal disease, hepatitis, or cancer were excluded from the study. Patients already receiving counseling for diabetes and hypertension were also not included.

### Sample Size and Sampling Technique

Eight community pharmacies located in twin cities were selected in the pre-intervention phase. A simple random sampling technique using the lottery method was used to select community pharmacies in groups A (intervention, *n* = 4) and group B (control, *n* = 4). A list of registered community pharmacists was obtained from the District Health Office of Islamabad and Rawalpindi (DHO), which helped to select community pharmacists randomly. Pharmacists working in these community pharmacies included in group A (intervention group) were trained, while no training was given to pharmacists working in community pharmacies included in group B (control group). Out of eight selected community pharmacies, the pharmacists working at these pharmacies who were willing to participate were included in the study. Out of eight pharmacists, six were males. In terms of qualification, six had Pharm. D degree while two had done M. Phil. in Pharmacy Practice. Moreover, the pharmacists had working experience as three had the experience of more than 5 years, four had the experience of 1 year, while one had the experience of 2 years. According to WHO, at least thirty encounters must be included in each group to assess the impact of the intervention. The total number of patients was 40 in each group, while estimating a dropout rate of 25%. The convenience sampling technique was used to select patients who visited community pharmacies. Ten patients were selected from each community pharmacy after taking their consent to participate in the study.

### Design and Implementation of the Development of the Intervention Material

The objectives, content and intervention format were designed after a series of discussions with different stakeholders. The training material content was developed from the International Diabetes Federation Diabetes Education Module (Unwin et al., 2010) and Pharmacy- Based Hypertension Management Model: Protocol and Guidelines (World Health Organization, 2005). The name of the training module was recommended as ‘Clinical Skills for the Management of Diabetes Mellitus and Hypertension'.

### General Description of Training

The community pharmacists were trained by the principal and co-investigators at community pharmacies.

1) Training Aids used

The pharmacists were also provided with brochures and one-pagers related to diabetes and hypertension management and counseling, glucose and blood pressure log sheets, glucometer, BP measuring devices, and questionnaires to assess disease knowledge and medication adherence. Pharmacists were also provided patient kits containing disease brochures, diet charts, BP, and glucose monitoring cards.

2) Post Training Data Collection

Patients in the intervention group received special counseling sessions by the community pharmacist, whereas those in the control group received the usual pharmacy services, i.e., dispensing medications and providing information regarding medication administration. Patients enrolled in the control and intervention group were required to visit the community pharmacy every 15 days for 6 months during the study. At enrollment, patients in the intervention group received counseling on the disease, its complications, medication, lifestyle modifications, and self-monitoring of the disease. Each patient also received consultations based on individual needs. Patient kits were provided along with counseling. The duration of counseling was a minimum of 20 min. Blood pressure and blood glucose were monitored for each patient in both control and intervention groups at each visit by community pharmacists using digital glucometers and mercury sphygmomanometers. Fasting blood glucose was measured on each visit. The patient was instructed to follow the 8-h fasting protocol prior to testing. HbA1c values were not mentioned as they were not directly performed at the community pharmacy setting; however, the pharmacist reviewed the HBa1c values carried out by an independent lab.

### Data Collection Tools

Pre-validated tools were used for the study. Written permission had been obtained from the respective organization. A pre-validated tool, Diabetes Knowledge Questionnaire 24 (Bukhsh et al., 2017), was used to assess diabetes knowledge. The Urdu version was utilized for this study. The questionnaire comprised 24 questions related to the etiology of diabetes, symptoms, lifestyle modifications, and complications. The scoring of the DKQ-24 included the sum of all correct items of each respondent. One point was given to each correct answer and no point for the incorrect option. The score range of the tool is 0–24, and the higher score indicates better patient knowledge regarding diabetes. The second prevalidated tool used for the assessment of hypertension knowledge was the Hypertension Knowledge Level Scale (Erkoc et al., 2012). The questionnaire comprised twenty-two questions related to the definition, drug compliance, lifestyle, diet, and complications. The HK-LS scoring included the sum of all correct items of each respondent. One point was given to each correct answer and no point for the incorrect option. The score range of the tool is 0–22. Higher scores indicate better patient knowledge about hypertension. Medication adherence to diabetes and hypertension medicine was assessed using the Brief Medication Questionnaire (Demoz et al., 2020). The tool BMQ is comprised of eleven questions divided into three screens according to the barriers faced by the patient: Regimen screen that asks patients about the administration of medication in the past week, a Belief screen that deals with questions related to effects of the drug and side effects and a recall screen comprised of questions related to remembrance of potential difficulties during the administration of medicines. The Brief Medication Questionnaire Adherence Risk Scale score ranges from 0 to 4, with “0” indicating no self-reported non-adherence or barriers to adherence and “4” indicating the presence of self-reported non-adherence and three types of barriers (belief or motivational barrier, recall barrier, and access barrier). A score of 1 is given in each question if the patient reports adherence to the current regimen. A score of 0 is given if the patient reports non-adherence to medications (Shehab et al., 2016). A score ≥1 indicates a positive screen for a particular barrier. Blood pressure and glucose log sheets were designed to monitor BP and glucose after every 15 days. The mean readings were calculated after 3 months and 6 months.

### Reliability of Tools

Pilot testing was conducted on 10% of the sample to check the reliability of all three tools. The Cronbach’s alpha value was found to be 0.813 for Hypertension Knowledge Level Scale, 0.80 for Diabetes Knowledge Questionnaire 24, and 0.761 for the Brief Medication Questionnaire, respectively.

### Data Collection Procedure and Data Analysis

Data were collected by community pharmacists trained by the principal and co-investigators. The questionnaires were administered by the pharmacists to the respondents at baseline and after 6 months. Selected community pharmacists monitored blood pressure and glucose after every 15 days for 6 months. After data collection, data were coded and entered in SPSS version-21. To check the distribution of the data, a skewness test was performed. Descriptive statistics comprising of frequency and percentages were calculated. Wilcoxon test (*p* < 0.05) was used to compare pre-post intervention knowledge regarding diabetes and hypertension. Mann-Whitney test (*p* < 0.05) was used to find differences among medication adherence among control and intervention groups and pre-and post-intervention.

## Results

### Demographic Characteristics

Of the 40 respondents to the control group, 57.5% (*n* = 23) were males, while 42.5% (*n* = 17) were females. Of the total respondents, 20% (*n* = 8) had diabetes for the past 1–3 years, whereas 10% (*n* = 4) had a history of diabetes for more than 6 years. The majority of the respondents had a history of hypertension for more than 6 years (32.5%, *n* = 13). However, among the respondents to the intervention group, 52.5% (*n* = 21) were men, while 47.5% (*n* = 19) were women. Out of the total respondents, 25% (*n* = 10) had diabetes for the past 1–3 years, whereas 7.5% (*n* = 3) had a history of diabetes for more than 6 years. The majority of the respondents had a history of hypertension for 1–3 years (37.5%, *n* = 15). A detailed description is given (Table 1).

**TABLE 1:** Respondents demographic characteristics.

**Demographic characteristics**	**Control group n (%)**	**Intervention group n (%)**
Age		
20–30 years	5 (12.5)	3 (7.5)
31–40 years	12 (30)	11 (27.5)
41–50 years	13 (32.5)	15 (37.5)
51–60 years	6 (15)	8 (20)
>60 years	4 (10)	3 (7.5)
Gender		
Male	23 (57.5)	21 (52.5)
Female	17 (42.5)	19 (47.5)
Level of Education		
Matric	3 (7.5)	5 (12.5)
Intermediate	5 (12.5)	3 (7.5)
Graduate	23 (57.5)	23 (57.5)
Postgraduate	4 (10)	6 (15)
Illiterate	5 (12.5)	3 (7.5)
Duration of Diabetes Mellitus		
<1 year	3 (7.5)	4 (10)
1–3 years	8 (20)	10 (25)
4–6 years	25 (62.5)	23 (57.5)
>6 years	4 (10)	3 (7.5)
Duration of Hypertension		
<1 year	4 (10)	3 (7.5)
1–3 years	8 (20)	15 (37.5)
4–6 years	15 (37.5)	12 (30)
>6 years	13 (32.5)	10 (25)

### Impact of Community Pharmacist Counseling on Knowledge of Patients With Diabetes and Hypertension in Intervention Group

The results of the present study showed that the mean knowledge scores regarding diabetes mellitus and hypertension among the control group at baseline were (13.95 ± 3.11) and (14.67, ± 2.48) respectively, which did not show any improvement after 6 months, i.e., (12.72 ± 3.47) and (14.32 ± 2.42). Mean knowledge scores regarding diabetes mellitus among intervention group at baseline was (16.02 ± 2.93) which was improved after 6 months to (19.97 ± 2.66). Although the mean knowledge scores regarding hypertension among the intervention group at baseline was 15.60 ± 3.33 which improved after 6 months to 18.35 ± 2.31. A detailed description is given (Table 2).

**TABLE 2:** Impact of community pharmacist counseling on knowledge of patients with diabetes and hypertension in intervention group.

Indicators	Diabetes mellitus				Hypertension			
	Baseline control	After 6 Months control	Baseline intervention	After 6 Months intervention	Baseline control	After 6 Months control	Baseline intervention	After 6 Months intervention
Mean^a^ (±SD)	13.95 (±3.11)	12.72 (±3.47)	16.02 (±2.93)	19.97 (±2.66)	14.67 (±2.48)	14.32 (±2.42)	15.60 (±3.33)	18.35 (±2.31)
Median	13.00	13.00	16.50	20.00	15.00	15.00	16.00	19.00

a Higher mean scores show better knowledge.

### Comparison of Pre-Post Intervention Knowledge of Patients With Diabetes Mellitus and Hypertension

Significant difference was observed (*p* ≤ 0.05) in pre-post intervention knowledge regarding diabetes and hypertension management. Knowledge of patients was improved regarding different aspects of diabetes and hypertension management after counseling by community pharmacists. A detailed description is given (Table 3).

**TABLE 3:** Comparison of pre-post intervention knowledge of patients with diabetes mellitus and hypertension.

**Knowledge**		**N**	**Mean rank**	**Sum of ranks**	**Diabetes knowledge**		**Hypertension knowledge**	
					**z-value**	**p-value**	**z-value**	**p-value**
Diabetes Knowledge	Negative Ranks	2	6.00	15.00	-5.109	**0.003**	-6.125	**0.002**
	Positive Ranks	36	19.50	525.00				
	Ties	2						
	Total	40						
Hypertension Knowledge	Negative Ranks	4	7.00	14.00				
	Positive Ranks	33	22.15	750.00				
	Ties	3						
	Total	40						

Bold values show Significant differences.

### Impact of Community Pharmacist Counseling on Blood Glucose and Blood Pressure Management at Baseline, 3 and 6 Months among Control and Intervention Group

The results of the study showed that the mean fasting blood glucose levels at baseline among control (11.60 ±1.50) and intervention (11.92 ±2.15) groups was quite similar. However, the fasting blood glucose level improved at 3 months (9.32 ±1.99) and after 6 months (8.25 ±1.48) in intervention group. At baseline, the mean systolic blood pressure between the control group (142.15 ±9.36) and the intervention group (145.85 ±10.88) did not show any significant differences. On the other hand, systolic BP decreased between the intervention group at 3 months (130.18 ±10.85) and 6 months (130.10 ±6.89). A detailed description is given (Table 4).

**TABLE 4:** Impact of community pharmacist counseling on blood glucose and blood pressure management at baseline, 3 and 6 months among control and intervention group.

Indicators	Control group			Intervention group		
	Baseline	Three Months	Six Months	Baseline	Three Months	Six Months
Fasting Blood Glucose (mmol/L)	11.60 (±1.50)	11.88 (±1.62)	11.54 (±1.56)	11.92 (±2.15)	9.32 (±1.99)	8.25 (±1.48)
Mean ± SD						
Systolic BP (mmHg)	142.15 (±9.36)	140.75 (±7.93)	145.48 (±6.69)	145.85 (±10.88)	130.18 (±10.85)	130.10 (±6.89)
Mean ± SD						
Diastolic BP (mmHg)	95.00 (±8.85)	96.50 (±8.36)	97.00 (±6.90)	95.08 (±10.96)	91.68 (±6.25)	88.83 (±5.38)
Mean ± SD						

### Impact of Community Pharmacist Counseling on Blood Glucose and Blood Pressure Management at Baseline, 3 and 6°Months according to Different Demographic Variables Intervention Group

The results of the present study highlighted that a reduction was observed in fasting blood glucose level, systolic and diastolic blood pressure among all demographic variables. Respondents aged 31–40 years showed a comparatively greater decrease in blood glucose from baseline (11.90 **±**2.38) after 3 months (9.38, **±**2.22) and 6 months (9.01, ±1.52). Moreover, respondents aged 31–40 years also showed comparatively greater decrease in systolic blood pressure from baseline (142.50, ±17.67) after 3 months (129, **±**12.72) and 6 months (122, **±**14.14). Male respondents had comparatively improved blood glucose level (8.87, ±1.47) and systolic blood pressure (127.22, ±7.11) after 6 months. A detailed description is given (Table 5).

**TABLE 5:** Comparison of the impact of community pharmacist counseling on the management of blood glucose level and blood pressure at baseline, 3 and 6 months according to different demographic variables between intervention group.

**Demographic variables**	**Outcome measures**	**Intervention group**		
		**Fasting Blood Glucose (mmol/L) Mean (±SD)**	**Systolic BP (mmHg) Mean (±SD)**	**Diastolic BP (mmHg) Mean (±SD)**
Age				
20–30 years	Baseline	11.07 (**±**2.46)	149.43 (**±**12.13)	99.00 (**±**5.65)
	3 Months	10.43 (**±**2.37)	137 (**±**9.55)	87.50 (**±**3.53)
	6 Months	10.00 (±1.99)	129 (**±**7.31)	87.50 (**±**3.53)
31–40 years	Baseline	11.90 (**±**2.38)	142.50 (**±**17.67)	99.14 (±8.27)
	3 Months	9.38 (**±**2.22)	129 (**±**12.72)	90 (**±**5.77)
	6 Months	9.01 (±1.52)	122 (**±**14.14)	83.57 (±4.75)
41–50 years	Baseline	11.52 (**±**1.65)	142.27 (**±**10.57)	98.64 (**±**9**.**66)
	3 Months	11.91 (**±**1.55)	131.91 (**±**10.44)	87.27 (**±**6.46)
	6 Months	11.54 (±1.06)	129.36 (**±**5.95)	86.73 (±4.10)
51–60 years	Baseline	11.05 (**±**2.47)	146.15 (**±**10.99)	98.70 (**±**8.64)
	3 Months	10.25 (**±**1.76)	131.15 (**±**11.36)	85.10 (**±**6.17)
	6 Months	10.10 (±1.55**)**	130.36 (**±**5.95)	85.10 (**±**6.17)
>60 years	Baseline	12.07 (**±**2.46)	152.15 (**±**10.99)	100.70 (**±**8.64)
	3 Months	11.43 (**±**2.37)	149.15 (**±**9.99)	99.70 (**±**7.69)
	6 Months	11.02 (±1.99)	149.15 (**±**9.99)	99.70 (**±**7.69)
Gender				
Male	Baseline	11.84 (±1.99)	146.07 (±11.15)	94.23 (±7.86)
	3 Months	9.23 (±1.86)	131.89 (±10.03)	86.92 (±6.304)
	6 Months	8.87 (±1.47)	127.22 (±7.11)	83.08 (±5.96)
Female	Baseline	11.09 (±2.23)	148.46 (±10.87)	96.04 (±8.77)
	3 Months	9.52 (±2.10)	133.08 (±12.33)	92.56 (±7.22)
	6 Months	9.12 (±1.45)	130.46 (±6.57)	92.56 (±7.22)
Level of Education				
Matric	Baseline	11.51 (±2.02)	147.60 (±10.15)	99.50 (±9.84)
	3 Months	8.99 (±1.89)	141.50 (±12.25)	96.20 (±4.82)
	6 Months	8.60 (±1.10)	141.50 (±12.25)	96.20 (±4.82)
Intermediate	Baseline	11.27 (±2.26)	145.00 (±13.69)	99.67 (±7.50)
	3 Months	9.53 (±2.14)	149.78 (±13.24)	99.67 (±7.50)
	6 Months	8.87 (±1.44)	146.11 (±6.00)	94.20 (±4.82)
Graduate	Baseline	11.85 (±2.36)	135.92 (±10.16)	89.54 (±8.52)
	3 Months	9.28 (±2.21)	132.15 (±8.18)	85.38 (±5.93)
	6 Months	9.06 (±1.78)	132.15 (±8.18)	85.38 (±5.93)
Postgraduate	Baseline	11.00 (±1.00)	140.50 (±11.25)	98.20 (±4.82)
	3 Months	8.00 (±1.20)	136.92 (±10.16)	90.54 (±8.52)
	6 Months	7.00 (±1.00)	127.45 (±6.88)	82.54 (±5.18)
Illiterate	Baseline	11.16 (±1.60)	140.86 (±11.76)	92.71 (±7.41)
	3 Months	10.59 (±1.56)	139.14 (±10.52)	92.71 (±7.41)
	6 Months	10.59 (±1.56)	139.14 (±10.52)	90.14 (±6.98)
Duration of Diabetes Mellitus				
1–3 years	Baseline	11.82 (±2.17)	147.67 (±10.52)	98.67 (±6.05)
	3 Months	9.17 (±1.98)	130.83 (±12.41)	81.67 (±4.08)
	6 Months	8.55 (±0.914)	129.17 (±7.36)	80.00 (±8.36)
4–6 years	Baseline	11.15 (±1.69)	144.00 (±11.91)	98.38 (±9.02)
	3 Months	8.50 (±1.52)	129.13 (±11.85)	85.00 (±4.62)
	6 Months	8.30 (±1.18)	125.00 (±8.01)	81.88 (±2.58)
>6 years	Baseline	11.24 (±2.15)	149.00 (±10.65)	94.11 (±7.43)
	3 Months	9.66 (±2.04)	134.68 (±9.18)	88.79 (±6.24)
	6 Months	9.22 (±1.62)	127.89 (±6.30)	83.95 (±5.15)
Duration of Hypertension				
1–3 years	Baseline	11.12 (±2.48)	143.20 (±10.06)	95.00 (±5.70)
	3 Months	10.32 (±2.41)	127.00 (±10.95)	85.00 (±5.00)
	6 Months	9.56 (±1.62)	126.00 (±4.18)	80.00 (±8.36)
4–6 years	Baseline	11.41 (±2.48)	147.29 (±10.95)	96.29 (±7.34)
	3 Months	8.80 (±2.30)	132.57 (±9.72)	85.71 (±6.07)
	6 Months	8.49 (±1.74)	124.29 (±8.38)	81.88 (±2.58)
>6 years	Baseline	11.69 (±1.84)	147.79 (±11.09)	98.00 (±9.48)
	3 Months	9.15 (±1.73)	132.88 (±11.00)	86.75 (±6.42)
	6 Months	8.81 (±1.28)	128.33 (±6.54)	83.95 (±5.15)

### Comparison of Medication Adherence Among Pre and Post Intervention Group of Diabetic and Hypertensive Patients

Significant difference (*p* ≤ 0.05) was observed in regimen screen, belief screen and recall screen of diabetes medication adherence among pre and post intervention groups. Moreover, significant difference (*p* ≤ 0.05) was observed in regimen screen and recall screen of hypertension medication adherence among pre and post intervention groups. No difference (*p* ≥ 0.05) was observed in access screen of diabetes medication adherence and access and belief screen of hypertension medication adherence among pre and post intervention group. A detailed description is given (Table 6).

**TABLE 6:** Comparison of pre and post intervention medication adherence among diabetic and hypertensive patients.

**Adherence indicators**	***n***	**Mean ranks**	**Sum of ranks**	**Test statistics**	**p-value**
Diabetes Medication Adherence					
Regimen Screen	Baseline: 40	Baseline: 48.03	Baseline: 1921.00	499.000	**0.001**
	Six months: 40	Six months: 32.98	Six months: 1319.00		
Belief Screen	Baseline: 40	Baseline: 21.45	Baseline: 858.00	38.000	**0.001**
	Six months: 40	Six months: 59.55	Six months: 2382.00		
Recall Screen	Baseline: 40	Baseline: 20.50	Baseline: 820.00	0.000	**0.001**
	Six months: 40	Six months: 60.50	Six months: 2420.00		
Access Screen	Baseline: 40	Baseline: 40.50	Baseline: 1620.00	800.000	0.568
	Six months: 40	Six months: 40.50	Six months: 1620.00		
Hypertension Medication Adherence					
Regimen Screen	Baseline: 40	Baseline: 48.79	Baseline: 1951.00	468.500	**0.001**
	Six months: 40	Six months: 32.98	Six months: 1288.50		
Belief Screen	Baseline: 40	Baseline: 43.36	Baseline: 1734.50	685.500	0.152
	Six months: 40	Six months: 37.64	Six months: 1505.50		
Recall Screen	Baseline: 40	Baseline: 45.05	Baseline: 1802.00	618.000	**0.051**
	Six months: 40	Six months: 35.95	Six months: 1438.00		
Access Screen	Baseline: 40	Baseline: 41.63	Baseline: 1665.00	800.000	0.568
	Six months: 40	Six months: 39.38	Six months: 1575.00		

Mann-Whitney Test (*p* ≥ 0.05).

Bold values show Significant differences.

## Discussion

The provision of pharmaceutical care plays an important role in achieving the specified goals for patients with hypertension as well as diabetes mellitus. Pharmaceutical care and medication management services provided by community pharmacists can improve blood pressure as well as blood glucose level and improve quality of life. The results of the present study showed that the knowledge regarding diabetes mellitus and hypertension improved over 6 months in the intervention group that received counseling from a community pharmacist. The post-intervention group had better knowledge regarding disease, medication use, and complications of diabetes and hypertension. Similar findings were reported in a study conducted in France where the knowledge regarding diabetes and hypertension improved after counseling by community pharmacists (Delage et al., 2021). Pharmaceutical care programs initiated by community pharmacists can improve blood pressure and glycemic goals (Venkatesan et al., 2012). A significant reduction was observed in fasting blood glucose in the intervention group compared to the control group after 3 and 6 months. The intervention group showed a significant decrease in systolic blood pressure as compared to the control group after 3 and 6 months. Furthermore, a significant reduction was observed in diastolic blood pressure among the intervention group. This might be because community pharmacists are qualified personnel in a better position to clarify/answer different misconceptions or queries of patients. Similar findings were reported by studies conducted in numerous countries, where pharmacist counseling led to improved blood glucose levels, blood pressure, and lipid control (McAlister et al., 2014; Butt et al., 2016; Lakey et al., 2020; Reeves et al., 2021). Males in the current study reported better post-intervention diabetes and hypertension control than females. Similar findings were reported in several countries including Egypt, where a significant improvement in diabetes and hypertension management was reported after community pharmacist counseling (Khalaf et al., 2019). Better qualification was identified as an important factor in the present study, which helped improve disease management after receiving counseling from a community pharmacist. This might be due to the fact that better qualification helps in better understanding of different facts and terminologies related to disease knowledge as it helps the patient to clarify concepts by asking more questions after receiving the counseling. Similar results were reported in a study conducted in India where patients having better qualifications reported improved diabetes and hypertension management after receiving counseling by community pharmacists (Venkatesan et al., 2012).

Community pharmacists have a major role in optimizing medication therapy and improving patient adherence (Fikri-Benbrahim et al., 2013). The results of the current study showed that medication adherence improved among patients with diabetes and hypertension. The majority of the patients started to take their medicines on time after 6 months in the intervention group. Similar results were reported by a study conducted in China where medication adherence was improved after receiving counseling by community pharmacists (Li et al., 2021). Moreover, the patients enrolled in the present study believed that the drug worked for them. The majority of them agreed that they remembered the doses of their medications for hypertension and diabetes and did not worry about the side effects of the drugs. Similar findings were reported in a study conducted in China where patient adherence was improved after community pharmacist intervention (Li et al., 2021).

The results of this study helped to affirm the role of community pharmacist as an integral member of primary care services to maximize the quality of provided care and to develop a framework for medication therapy management for chronic diseases such as diabetes and hypertension that matches the specific needs of the population. The development and implementation of the current community pharmacist-led diabetes and hypertension program provide robust evidence for the stakeholders to further develop various community pharmacist-led innovative services in Pakistan.

## Limitations

A few of the limitations faced during the conduction of this study included time and financial constraints. The respondents were followed up for only 6 months, so the long-term benefits of this community pharmacy model could not be observed at length. The study findings may not be generalized to other parts of the country as it was conducted within twin cities of Pakistan.

## Conclusion

The current study proved the effectiveness of the pilot model of community pharmacy Diabetes and Hypertension Care Program implemented in twin cities of Pakistan. The study concluded that community pharmacist counseling had a positive impact on diabetes and hypertension management as it has helped patients in achieving their desired blood pressure and blood glucose goals, including improvement in their medication adherence. Better health literacy and socioeconomic status can also help perceive counseling positively, leading to better diabetes and hypertension management. The effectiveness of the pilot model suggests that pharmacist-led diabetes and hypertension management programs must be initiated at community pharmacies across the country where pharmacists can provide education regarding disease, its management and self-care activities.

## Data Availability

The raw data supporting the conclusions of this article will be made available by the authors, without undue reservation.
